# Goat-derived hyperimmune colostrum and milk following vaccination with live-attenuated and inactivated porcine epidemic diarrhea virus: Safety and immunogenicity evaluation

**DOI:** 10.14202/vetworld.2025.2689-2698

**Published:** 2025-09-11

**Authors:** Poonnika Suvannabha, Pimpakarn Suwan, Alongkot Boonsoongnern, Niorn Ratanapob, Yonlayong Woonwong, Manakorn Sukmak, Prapassorn Boonsoongnern

**Affiliations:** 1 Program of Animal Health and Biomedical Sciences, Faculty of Veterinary Medicine, Kasetsart University, Bangkok 10900, Thailand; 2 Department of Farm Resources and Production Medicine, Kasetsart University, Kamphaeng Saen Campus, Nakhon Pathom 73140, Thailand; 3 Department of Large Animal and Wildlife Clinical Science, Kasetsart University, Kamphaeng Saen Campus, Nakhon Pathom 73140, Thailand; 4 Department of Anatomy, Faculty of Veterinary Medicine, Kasetsart University, Bangkok 10900, Thailand

**Keywords:** goat colostrum, hyperimmune milk, neutralizing antibody titer, passive immunity, porcine epidemic diarrhea virus, vaccine safety

## Abstract

**Background and Aim:**

Porcine epidemic diarrhea virus (PEDV) is a highly contagious enteric pathogen causing severe diarrhea and high mortality in neonatal piglets. Maternal lactogenic immunity, conveyed through colostrum and milk, is essential for protection; however, sow-derived antibodies may be insufficient in certain production systems. This study aimed to produce PEDV-specific hyperimmune colostrum and milk from goats and evaluate the safety and immunogenicity of live-attenuated and inactivated PEDV vaccines.

**Materials and Methods:**

Preliminary safety trials were performed in male goats (n = 6) to monitor clinical signs and adverse reactions after intramuscular vaccination. Ten pregnant Saanen goats were randomly assigned to two groups (n = 5 each) and immunized twice, 8 and 4 weeks before parturition, with either live-attenuated (1 × 10^5^ 50% tissue culture infectious dose [TCID_50_]/mL) or inactivated (1 × 10^6^ TCID_50_/mL) PEDV vaccine. Serum was collected on days 0 and 28 post-vaccination, and colostrum/milk samples were obtained on days 0, 2, 7, and 14 postpartum for virus neutralization (VN) assays. Fecal samples were analyzed using quantitative reverse transcription polymerase chain reaction to detect viral shedding.

**Results:**

Both vaccines were well tolerated, with only transient fever observed in three goats. No severe adverse reactions occurred in pregnant goats. The live-attenuated vaccine elicited higher VN titers in serum (peak 1:32) and colostrum (peak 1:2048) compared with the inactivated vaccine (serum peak 1:16; colostrum peak 1:512). Day 0 colostrum titers were significantly greater in the live vaccine group (p = 0.028). Although titers remained higher in this group on days 2, 7, and 14, differences were not statistically significant. Viral RNA shedding was absent in the inactivated group and transient in the live group, persisting up to 7 days in one goat.

**Conclusion:**

Vaccination of pregnant goats with live-attenuated or inactivated PEDV vaccines is safe and induces PEDV-specific antibodies in colostrum and milk. The live-attenuated vaccine generated the highest titers, indicating potential for producing goat-derived hyperimmune colostrum as an alternative passive immunization strategy for neonatal piglets. Field trials are warranted to confirm protective efficacy and explore integration into PEDV control programs.

## INTRODUCTION

Porcine epidemic diarrhea virus (PEDV), a member of the genus *Alphacoronavirus* within the family *Coronaviridae* and order *Nidovirales*, was first reported in England [1] and Belgium [2] in the late 1970s. Since then, PEDV has spread across several Asian countries, including Thailand, where it caused widespread outbreaks and eventually became endemic [3–7]. More recently, outbreaks have been reported in India, underscoring the continued expansion of the virus across Asia [8]. PEDV causes acute diarrhea, vomiting, and dehydration in pigs of all ages; however, neonatal piglets are particularly susceptible, with mortality rates approaching 100% due to the absence of innate immunity and the slower turnover of villous enterocytes [9]. This delayed turnover hampers the proliferation of intestinal stem cells compared with that in weaned pigs [10], resulting in severe clinical outcomes. PEDV infections in piglets contribute to substantial economic losses in the global swine industry [11], making the prevention of clinical disease in piglets a critical priority.

Because of the epitheliochorial structure of the sow placenta, newborn piglets are born agammaglobulinemic and immunosuppressed, relying entirely on colostrum and milk for passive immunity until weaning [12, 13]. Passive lactogenic immunity against viruses such as PEDV, transmissible gastroenteritis virus, and porcine deltacoronavirus remains the most effective strategy for protecting neonatal piglets from enteric viral infections [14]. However, inadequate colostrum intake can impair maternal antibody transfer, increasing piglet susceptibility to infection [15]. Ensuring adequate colostrum consumption is therefore essential for neonatal health and immune protection. When natural intake is insufficient, supplemental feeding strategies become necessary. Martínez Miró *et al*. [16] demonstrated that piglets can absorb immunoglobulin (Ig) G from goat colostrum within the first 12 h postpartum, achieving an absorption efficiency of 20.9%. Notably, no diarrhea or intolerance was observed, and goat colostrum was well tolerated within the 1^st^ h of administration.

Goat colostrum is rich in IgG and whey proteins containing bioactive peptides, recognized for their anti-inflammatory, immunomodulatory, and gut-enhancing properties [17–19]. These characteristics make goat colostrum a promising supplemental source of passive immunity for newborn piglets. A previous by Shibata *et al*. [20] has shown that hyperimmune cow colostrum (HCC) with a PEDV antibody titer of 1:512 can effectively prevent infection and reduce piglet mortality, while IgY antibodies from chicken egg yolk have similarly reduced mortality rates [21]. Compared with conventional sow vaccination or colostrum replacers, producing PEDV-specific hyperimmune colostrum in goats offers a novel, flexible alternative, particularly beneficial where sow-derived immunity is limited, and allows production independent of swine herds, thereby enhancing biosafety and scalability.

In addition to its immunological advantages, goat colostrum has favorable nutritional properties. Ayala *et al*. [22] reported that its fatty acid profile is well-suited for neonatal piglet diets, with short- and medium-chain saturated fatty acid levels approximately three times higher than those in swine colostrum, potentially improving early survival. Furthermore, goat milk contains smaller fat globules than cow milk, increasing surface area and enhancing digestibility [23].

While sow vaccination remains the primary strategy to confer lactogenic immunity against PEDV, its effectiveness can be inconsistent, particularly in herds experiencing early-stage or recurrent infections. In hyperprolific sows, maternal antibodies may not be uniformly distributed among large litters, leaving some piglets inadequately protected. Moreover, the production of PEDV-specific hyperimmune colostrum is largely limited to swine herds, which may pose biosafety challenges and limit scalability in PEDV-endemic regions. Alternative passive immunization sources, such as HCC and IgY from chicken egg yolk, have shown promise, but cross-species Ig delivery in swine remains underexplored. Goats present an attractive alternative due to their manageable size, lower maintenance costs, and the bioactive and nutritional properties of their colostrum. Although goat colostrum has been proven safe and absorbable by piglets, no published studies have systematically evaluated the safety and immunogenicity of using goats vaccinated with PEDV to generate high-titer, pathogen-specific colostrum and milk. Furthermore, comparative data on the antibody response induced by live-attenuated versus inactivated PEDV vaccines in goats are lacking, as is information on viral shedding risks associated with live vaccines in non-susceptible host species. This gap limits the evidence base for implementing goat-derived hyperimmune milk as a scalable adjunct to PEDV control programs.

This study aimed to evaluate the safety and immunogenicity of two PEDV vaccine formulations, live attenuated and inactivated, when administered to goats during late gestation. Specifically, the objectives were (1) to assess the occurrence of adverse reactions and viral shedding in vaccinated goats; (2) to quantify PEDV-specific neutralizing antibody titers in serum, colostrum, and milk at multiple postpartum time points; and (3) to compare the antibody responses between the two vaccine formulations. By addressing these objectives, the study sought to determine the feasibility of using vaccinated goats as a novel source of hyperimmune colostrum and milk for passive immunization of neonatal piglets, thereby providing an alternative or supplementary strategy to conventional sow-based immunity in PEDV control programs.

## MATERIALS AND METHODS

### Ethical approval

All animal procedures were reviewed and approved by the Institutional Animal Care and Use Committee of the Faculty of Veterinary Medicine, Kasetsart University, Thailand (Approval No. ACKU64-VET-061). The study was conducted in accordance with the Animal Research: Reporting of *In Vivo* Experiments (ARRIVE) 2.0 guidelines for reporting *in vivo* animal experiments.

### Study period and location

The study was conducted from June 2022 to March 2024 at the Faculty of Veterinary Medicine, Kasetsart University, Kamphaeng Saen Campus, Nakhon Pathom, Thailand. Goats were housed in an open-sided shelter with a roof, allowing for natural ventilation. The facility provided adequate space, dry bedding, and shade to protect animals from direct sunlight and rain. Animals had *ad libitum* access to clean water and were managed under standard husbandry protocols.

### PEDV strain and vaccine preparation

The PEDV TRANG/37 strain (GenBank accession no. MN379926), isolated from the feces of an infected pig in Thailand, was propagated in Vero cells as described by Boonsoongnern *et al*. [24].

- Live-attenuated vaccine: Generated by 150 serial passages of PEDV-infected Vero cells. The final formulation contained 1 × 10^5^ 50% tissue culture infectious dose (TCID_50_)/mL attenuated PEDV with 10% Montanide Gel 01 adjuvant (Seppic, Seoul, Korea)
- Inactivated vaccine: Prepared from PEDV-infected Vero cells passaged 20 times and inactivated with binary ethylenimine at 37°C for 48 h. The inactivated antigen (1 × 10^6^ TCID_50_/mL) was formulated with Montanide Gel 01 adjuvant.

### Vaccine safety assessment in male goats

Six one-year-old male Saanen goats were randomly allocated into two groups (n = 3 per group). One group received the live-attenuated PEDV vaccine, and the other received the inactivated PEDV vaccine through intramuscular injection. Clinical signs and adverse reactions were monitored at 0, 3, 24, and 48 h post-vaccination.

### Experimental design and vaccination in pregnant goats

Ten pregnant Saanen goats were randomly assigned to two treatment groups (n = 5 per group) and housed in the same facility. Pregnancy was confirmed at 45–60 days of gestation through transabdominal ultrasonography (HS-1600V, Honda, Aichi, Japan).

- Group 1: Two intramuscular doses of live-attenuated PEDV vaccine (1 × 10^5^ TCID_50_/mL)
- Group 2: Two intramuscular doses of inactivated PEDV vaccine (1 × 10^6^ TCID_50_/mL)

Each goat received 2 mL of vaccine at 8 and 4 weeks before expected parturition. Goats were observed daily for 7-day post-vaccination (dpv) for rectal temperature, behavior, appetite, water intake, injection site reactions (swelling, heat, pain, abscess), and systemic abnormalities.

### Sample collection and storage

#### Blood sampling

Blood (10 mL) was collected from the jugular vein using an 18-gauge (1.20 × 25 mm) needle and syringe, transferred into BD vacutainer serum tubes (Becton, Dickinson and Company, Franklin Lakes, NJ, USA), centrifuged at 1,500 × *g* for 5 min at 25°C, and the serum was stored at −80°C. Samples were collected on day 0 (pre-vaccination) and day 28 post-vaccination for virus neutralization (VN) assays.

#### Colostrum and milk sampling

Samples were collected on day 0 (kidding) and days 2, 7, and 14 postpartum. After teat disinfection with 70% ethanol and discarding the first streams, ~15 mL was collected into sterile tubes, chilled on ice, centrifuged twice at 17,000 × *g* for 10 min at 4°C to remove fat, and stored at −80°C.

#### Fecal sampling

Feces were collected rectally into screw-cap tubes on day 0 (pre-vaccination) and days 2, 7, and 14 post-vaccination. Viral RNA was analyzed through quantitative reverse transcription polymerase chain reaction (qRT-PCR).

### Quantification of PEDV RNA by qRT-PCR

One gram of feces was diluted in 1 mL of distilled water, homogenized, and centrifuged. A 150 μL aliquot of supernatant was used for RNA extraction (phenol–chloroform method). Reverse transcription was performed using the RevertAid First Strand complementary DNA (cDNA) Synthesis Kit (Thermo Fisher Scientific, USA).

qRT-PCR targeted the PEDV M gene using the following:

- Forward primer: 5′-CAG GAC ACA TTC TTG GTG GTC TT-3′
- Reverse primer: 5′-CAA GCA ATG TAC CAC TAA GGA GTG TT-3′
- Probe: 5′-ACG CGC TTC TCA CTA C-3′.

Reactions contained 10 μL Luna Universal Probe quantitative polymerase chain reaction Master Mix (New England Biolabs, Ipswich, MA, USA), 0.4 μL probe, 1.6 μL primers, 2 μL cDNA, and nuclease-free water. Cycling: 95°C for 2 min, followed by 40 cycles of 95°C for 10 s and 60°C for 30 s. Viral RNA concentrations were determined from a standard curve prepared from *in vitro*-transcribed PEDV RNA. Samples with a cycle threshold ≥ 35 were classified as negative.

### VN assay

Samples were heat-inactivated at 56°C for 30 min and stored at −20°C. Colostrum/milk was centrifuged at 9,000 × *g* for 5 min at 4°C, diluted 1:2 in Iscove’s Modified Dulbecco’s Medium (IMDM, Thermo Fisher Scientific, USA), and filtered through 0.45 μm membranes.

PEDV TRANG/37 was mixed with processed samples and incubated at 37°C for 1 h, then added to Vero cell monolayers in 96-well plates. After washing with phosphate-buffered saline (pH 7.2), cells were maintained in IMDM with trypsin and incubated for 3 days. Cytopathic effects (CPEs) were assessed daily. The highest reciprocal dilution that completely inhibited CPE in duplicates was recorded as the VN titer, with ≥1:2 considered positive.

### Statistical analysis

Neutralizing antibody titers were log_2_-transformed. Data are expressed as mean ± standard deviation. Normality was assessed using the Shapiro–Wilk test. Group comparisons at each time point were performed using unpaired Student’s t-tests for parametric data or Mann–Whitney U tests otherwise. Statistical significance was set at p < 0.05. Analyses were performed using R software (v4.3.3; R Foundation for Statistical Computing, Vienna, Austria).

## RESULTS

### Vaccine safety assessment

Within 24 h post-vaccination, three goats developed fever. Two cases occurred in the live-attenuated PEDV vaccine group and one case occurred in the inactivated vaccine group. Recorded body temperatures ranged from 101°F to 104°F, consistent with the fever range defined by Abdisa [25] (Table 1). Each febrile goat received a 3 mL dose of Butasyl (Zoetis, Madrid, Spain) to reduce fever. Following treatment, temperatures returned to normal, and no further clinical signs were observed. No adverse effects were noted in pregnant goats after vaccination, and throughout the observation period, all pregnant goats remained healthy with no signs of illness or discomfort.

**Table 1:** Body temperatures (°F) of male goats 1 day after vaccination.

Vaccine	Goat’s code	Body temperature (°F)	Noted
Live-attenuated PED vaccine	1	105.7	Fever
	2	105.7	Fever
	3	103.6	Normal
Inactivated PED vaccine	1	103.0	Normal
	2	105.2	Fever
	3	103.2	Normal

PED = Porcine epidemic diarrhea

### Fecal PEDV RNA shedding

At 2 dpv, viral RNA was undetectable in goats L1, L3, and L4 of the live-attenuated PEDV vaccine group. However, shedding was detected in L2 and L5 at 2 days, with L5 continuing to shed virus at 7 dpv. By day 7, no viral RNA was detected in the remaining goats of the live vaccine group. In contrast, no viral shedding was observed at any time point in pregnant goats vaccinated with the inactivated PEDV vaccine (Table 2).

**Table 2:** Viral shedding in goats after vaccination with live-attenuated and inactivated PEDV vaccines (cut-off Ct = 35.00).

Vaccine	Goat’s code	2 days after the injection	7 days after the injection
Live-attenuated PED vaccine	L1	Negative	Negative
	L2	33.84	Negative
	L3	Negative	Negative
	L4	Negative	Negative
	L5	34.45	34.57
Inactivated PED vaccine	K1	Negative	Negative
	K2	Negative	Negative
	K3	Negative	Negative
	K4	Negative	Negative
	K5	Negative	Negative

PED = Porcine epidemic diarrhea, PEDV = Porcine epidemic diarrhea virus, Ct = Cycle threshold

### Serum anti-PEDV antibody responses

**Figure 1:**
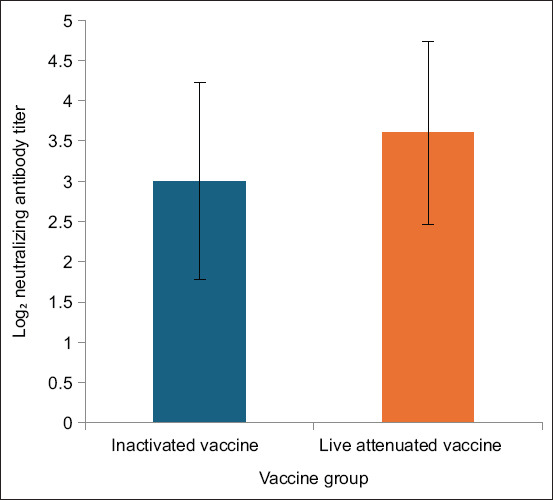
Serum log_2_ neutralizing antibody titers against porcine epidemic diarrhea virus in pregnant goats on day 28 vaccinated with live-attenuated or inactivated porcine epidemic diarrhea vaccine. Bars represent the mean ± standard deviation of log_2_-transformed neutralizing antibody titers in each vaccine group (n = 5 goats per group).

Neutralizing antibody levels in serum were evaluated by comparing pre- and post-vaccination samples. As shown in Table 3 and Figure 1, goat L3 in the live-attenuated group exhibited the highest serum titer (1:32). In the inactivated group, the maximum titer was 1:16, observed in goats K2 and K4. These results indicate that the live-attenuated vaccine induced a stronger humoral immune response than the inactivated vaccine. Although both groups developed detectable antibody titers, the difference between groups was not statistically significant (p > 0.05).

**Table 3:** Serum neutralizing titers of PEDV in goat serum samples.

Vaccine	Goat’s code	Antibody titer	Log_2_ titer
Live-attenuated PED vaccine	L1	1:8	3
	L2	1:16	4
	L3	1:32	5
	L4	1:16	4
	L5	1:4	2
Inactivated PED vaccine	K1	1:8	3
	K2	1:16	4
	K3	1:2	1
	K4	1:16	4
	K5	1:8	3

PED = Porcine epidemic diarrhea, PEDV = Porcine epidemic diarrhea virus

### Antibody titers in colostrum and milk

**Figure 2:**
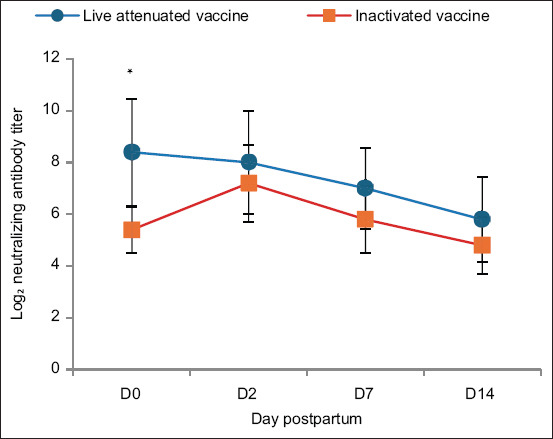
Time-course of log_2_-transformed neutralizing antibody titers in goat colostrum and milk collected on days 0, 2, 7, and 14 postpartum from goats vaccinated with either live-attenuated or inactivated porcine epidemic diarrhea virus vaccine. Data are presented as mean ± standard deviation (n = 5 per group). p < 0.05 indicates a statistically significant difference between groups on day 0.

Colostrum and milk neutralizing antibody titers were determined on days 0 (kidding), 2, 7, and 14 postpartum (Table 4). In the live-attenuated vaccine group, day 0 colostrum titers ranged from 1:64 (L1) to 1:2048 (L4). Although titers declined over time, goat L5 retained a titer of 1:512 on day 2. In the inactivated vaccine group, the highest day 0 titer was 1:128 (K5), with subsequent peaks of 1:256 in K2 (day 2) and 1:512 in K3 (day 7). Overall, the live-attenuated vaccine group produced more robust and sustained colostrum and milk antibody titers, indicating greater potential for prolonged passive immunity in neonates. Figure 2 provides a visual summary of these results.

**Table 4:** Neutralizing antibody titers and log_2_-transformed values in colostrum and milk samples.

Vaccine	Goat’s code	Day 0	log_2_ (D0)	Day 2	log_2_ (D2)	Day 7	log_2_ (D7)	Day 14	log_2_ (D14)
Live-attenuated PED vaccine	L1	1:64	6	1:32	5	1:32	5	1:16	4
	L2	1:1024	10	1:512	9	1:64	6	1:32	5
	L3	1:128	7	1:128	7	1:128	7	1:32	5
	L4	1:2048	11	1:1024	10	1:512	9	1:256	8
	L5	1:256	8	1:512	9	1:256	8	1:128	7
Inactivation of the PED vaccine	K1	1:32	5	1:128	7	1:64	6	1:8	3
	K2	1:32	5	1:256	8	1:32	5	1:32	5
	K3	1:32	5	1:512	9	1:256	8	1:64	6
	K4	1:32	5	1:32	5	1:32	5	1:32	5
	K5	1:128	7	1:128	7	1:32	5	1:32	5

PED = Porcine epidemic diarrhea

### Comparison of colostrum antibody titers

**Figure 3:**
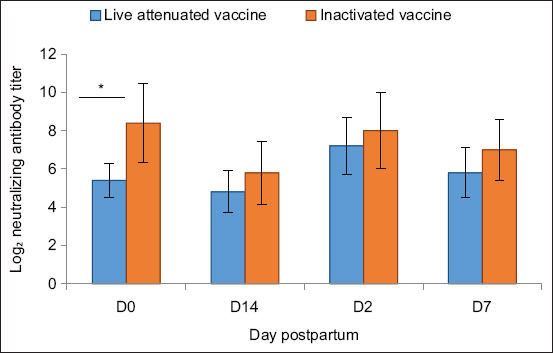
Neutralizing antibody titers (Log_2_) in colostrum and milk samples of pregnant goats at different postpartum time points. Bars represent the mean ± standard deviation of log_2_-transformed neutralizing antibody titers on days 0, 2, 7, and 14 for both live-attenuated and inactivated porcine epidemic diarrhea vaccine groups (n = 5 goats per group). Asterisks indicate statistically significant differences between groups at individual time points (p < 0.05).

A statistically significant difference in colostrum antibody titers between the two vaccine groups was observed only on day 0 (p = 0.028), with the live-attenuated vaccine group showing higher titers (Figure 3). While the live vaccine group maintained higher mean titers on days 2, 7, and 14, these differences were not statistically significant (p > 0.05).

## DISCUSSION

### Safety profile of vaccine formulations

The absence of severe adverse effects in vaccinated goats supports the safety of both the live-attenuated and inactivated PEDV vaccines. Mild fever was observed in three goats 1 dpv, possibly reflecting an early innate immune response. Such transient fevers are often associated with activation of natural killer cells, typically occurring within 1–2 days after antigen exposure [26]. The vaccines likely acted as antigenic stimuli, with the live-attenuated PEDV vaccine, carrying a higher antigenic load, designed to elicit a strong immune response [27]. Notably, two of the three febrile goats were in the live-attenuated vaccine group, reinforcing the association between vaccine replication potential and transient febrile reactions.

### Fecal viral shedding patterns

No fecal viral shedding was detected in goats vaccinated with the inactivated PEDV vaccine, consistent with its non-replicating nature. In contrast, viral RNA was detected in the live-attenuated vaccine group at 2 dpv, indicating the presence of a weakened but replicative virus [28]. This observation aligns with previous findings in piglets, where viral shedding persisted for 4–5.3 days after intramuscular administration [29, 30]. Variability in shedding duration, such as the prolonged excretion up to 7 days in goat L5, may be influenced by host-related factors, including age and underlying health status [9]. Additionally, shared housing could have facilitated horizontal transmission through the fecal–oral route [31]. These results confirm the safety of both vaccines but also highlight the potential risk of viral shedding from the live-attenuated formulation in group-housed environments.

### Serum neutralizing antibody responses

VN testing revealed a maximum serum titer of 1:32 in the live-attenuated group (L3) compared with 1:16 in the inactivated group (K2 and K4). This pattern is consistent with the known immunogenic profile of live-attenuated vaccines, which generally produce stronger immune responses and require fewer boosters than inactivated vaccines [28]. Comparable results were reported by Sato *et al*. [31], who observed serum titers of 1:32 and 1:128 in sows after two doses of a live-attenuated PEDV vaccine.

### Colostrum and milk antibody titers

High neutralizing antibody titers were detected in both vaccine groups. The peak colostrum titer of 1:2048 (L4, day 0) in the live-attenuated group far exceeded the average titer of 1:128 typically reported for sow colostrum, which is associated with reduced piglet mortality [32]. These results indicate that vaccinated goats can mount a strong lactogenic immune response, potentially surpassing that of sows. Unlike sow vaccination, this strategy enables production of high-titer, pathogen-specific colostrum in goats, facilitating potential cross-species immunization. The highest VN titer recorded (1:2048) reflects a robust individual response, highlighting the potential of goat-derived hyperimmune colostrum as a biosafe and scalable tool for PEDV control in endemic areas.

### Feasibility and practical considerations

Goats are cost-effective, easy to manage, and widely integrated into dairy systems, making them suitable for large-scale hyperimmune colostrum production. Vaccinations were strategically timed at 8 and 4 weeks before kidding to coincide with peak antibody production, which typically occurs 4–5 weeks before parturition [32]. For comparison, Shibata *et al*. [20] reported titers of 1:256–1:512 in cows vaccinated twice with inactivated PEDV, which were lower than the titers obtained in this goat study.

### Decline in antibody titers and potential applications

As expected, colostrum antibody titers declined over time, likely due to the transition to mature milk within the first 6 postpartum days, accompanied by reductions in fat, protein, and immune components. While sow-derived lactogenic immunity remains essential for protecting piglets, it may be inadequate in hyperprolific sows, where not all piglets receive sufficient antibodies, especially during early or active PEDV outbreaks. Supplementation with hyperimmune goat colostrum or milk could provide an effective adjunct strategy for enhancing passive immunity. Although piglets can absorb goat IgG without adverse effects, species-specific immune compatibility and compliance with regulatory requirements must be addressed before field application.

## CONCLUSION

This study demonstrated that both live-attenuated and inactivated PEDV vaccines are safe for administration in goats, with only transient fever observed in three animals and no severe adverse effects in either male or pregnant goats. The live-attenuated vaccine consistently induced higher serum, colostrum, and milk neutralizing antibody titers, reaching a peak colostrum titer of 1:2048, compared with the inactivated vaccine, which achieved a maximum colostrum titer of 1:512. Viral RNA shedding was absent in the inactivated group but transiently detected in the live vaccine group, persisting for up to 7 days in one goat. These findings confirm that goats can mount robust lactogenic immune responses to PEDV vaccination, producing high-titer, pathogen-specific colostrum and milk.

The results highlight the potential for using vaccinated goats as an alternative source of hyperimmune colostrum or milk for passive immunization of neonatal piglets, particularly in PEDV-endemic regions or in herds where sow-derived immunity is insufficient. This approach could be integrated into biosecurity protocols as a scalable, off-site Ig production system, reducing dependence on swine herd vaccination and improving supply resilience.

This work offers several strengths, including the first comparative evaluation of live-attenuated versus inactivated PEDV vaccines in goats, demonstration of high-titer PEDV-specific antibodies in goat colostrum exceeding titers typically reported in sow colostrum, and a strategic vaccination schedule optimized for peak antibody transfer at parturition. However, certain limitations should be acknowledged, such as the absence of challenge trials to confirm protective efficacy in piglets fed goat-derived hyperimmune colostrum, a limited sample size that may affect statistical power, and viral shedding data restricted to 14 dpv.

Future research should include controlled challenge studies to assess protection and survival rates in neonatal piglets supplemented with goat hyperimmune colostrum, evaluation of large-scale production feasibility, storage stability, and Ig persistence under farm conditions, and investigation of cross-protection potential against emerging PEDV variants and related coronaviruses.

Goat-based hyperimmune colostrum production represents a novel, feasible, and potentially transformative adjunct to PEDV control strategies. While the live-attenuated vaccine offers superior immunogenicity, its limited shedding risk requires careful housing and management. With further validation, this approach could enhance neonatal protection, reduce PEDV-associated mortality, and provide a flexible immunological safeguard for swine production systems worldwide.

## DATA AVAILABILITY

All the generated data are included in the manuscript.

## AUTHORS’ CONTRIBUTIONS

PB, AB, NR, and YW: Conceptualized the study. PS and PSw: Methodology. PS, PSw, and MS: Investigation and data analysis. PS: Writing – original draft. PB and AB: Reviewed and edited the manuscript. PB: Supervised the study and revised the manuscript. All authors have read and approved the final version of the manuscript.
